# Supplementary material for: Rapidly progressive arthropathy identified on imaging of patients treated with Crizotinib for ALK-rearranged/ROS1-positive non small cell lung cancer: A retrospective single-center study
Source: PLoS One. 2026 Feb 18;21(2):e0333223. doi: 10.1371/journal.pone.0333223 (PMC12915974; doi:10.1371/journal.pone.0333223)
Supplement: Attachment — Submitted filename: Response to the Editor.docx [file pone.0333223.s003.docx]

Dear Dr. Alejandro Torrado Pacheco,

Staff Editor

PLOS ONE

Thank you for the opportunity to submit a revised draft of our manuscript titled

“Rapidly progressive arthropathy identified on imaging of patients treated with

Crizotinib for ALK-rearranged/ROS1-positive non-small cell lung cancer: a

retrospective single-center study” to PLOS ONE.

We have incorporated changes to address the editors’ suggestions.

The changes have been highlighted within the manuscript.

Here is a point-by-point response to the editors’ comments and concerns.

Comment 1:

In your Methods section, you state that the image evaluators had access to identifying information. Could you please clarify what is meant by this?

Response:

Thank you for the chance to clarify this point. The image evaluators used the patient’s real identification number to locate the study on the picture archiving and communication system (PACS) system, and they could also see the dates of the study, but they were unaware of the patient’s clinical details, specifically whether the patients received Crizotinib or whether they were symptomatic. We changed in the text:

“The readers … were blinded they their clinical symptoms and whether they were treated with crizotinib”.

Comment 2:

Related to the above, could you please confirm whether this was approved by your IRB, and whether informed consent was waived? It would be helpful to upload a full English translation of your IRB approval letter for the editor and reviewers to assess. This does not need to be an official translation.

Response:

In the methods section it is stated:

“Approval from an institutional review board was obtained for the retrospective assessment of the patient’s clinical and radiological records. The patient’s informed consent was waived due to the study's retrospective nature.”

I have uploades a full English translation of our IRB approval letter, as well as confirmation of approval by the Institutional Review Board.

Comment 3:

Given that identifying information was available, this can introduce bias by unblinding the image evaluators to the treatment group (crizotinib vs no crizotinib) during image assessment. Please acknowledge this limitation in your manuscript.

Response:

The image evaluators were unaware of the treatment group during image assessment.

Comment 4:

Please report a confidence limit for the result that arthropathy was present in 18% of crizotinib-treated patients.

Response:

I added Confidence limit to the abstract, as it was already included in the results section.

Comments 5+6

Given the very small sample size for this study and the long period of observation, please take care not to overstate the conclusions. Please remove the sentence " There is a higher incidence of progressive arthropathy in NSCLC patients treated with crizotinib" from the abstract, or rephrase it to temper the conclusions given the setting and sample size of the study.

In general, it would be advisable to be less conclusive with this study's findings throughout, given the limitations noted above.

Response:

I sincerely apologize for any oversight in ensuring consistency across the manuscript and the editorial manager system. The sentence in question has already been revised in the updated version of the manuscript to: “We found progressive arthropathy, mostly painless, in one or more joints or intervertebral spaces of patients receiving crizotinib for NSCL”, but I forgot to change it in the editorial manager system.

I appreciate your thoughtful guidance regarding the importance of tempering conclusions, particularly given the small sample size and extended observation period. I will make sure to update the abstract within the editorial manager system to reflect the revised wording.
