# Supplementary material for: Rapidly progressive arthropathy identified on imaging of patients treated with Crizotinib for ALK-rearranged/ROS1-positive non small cell lung cancer: A retrospective single-center study
Source: PLoS One. 2026 Feb 18;21(2):e0333223. doi: 10.1371/journal.pone.0333223 (PMC12915974; doi:10.1371/journal.pone.0333223)
Supplement: Attachment — Submitted filename: Response to the Editor Dec. 2025.docx [file pone.0333223.s004.docx]

Dear Dr. Alejandro Torrado Pacheco,

Staff Editor

PLOS ONE

Thank you for the opportunity to submit a revised draft of our manuscript titled

“Rapidly progressive arthropathy identified on imaging of patients treated with

Crizotinib for ALK-rearranged/ROS1-positive non-small cell lung cancer: a

retrospective single-center study” to PLOS ONE.

We have incorporated changes to address the editors’ suggestions.

The changes have been highlighted within the manuscript.

Here is a point-by-point response to the editors’ comments and concerns.

Comment 1:

Discussion paragraph 1: "Such findings were not found in patients receiving other TKI inhibitors for NSCLC, implying that this is not a class effect but rather a drug-specific effect." > temper this statement as it cannot be supported by the data. At most this "may be" a drug-specific effect.

Response:

We changed in the text:

“Such findings were not found in patients receiving other TKI inhibitors for NSCLC, implying that this is not a class effect but rather may be a drug-specific effect.”

Comment 2:

Discussion last paragraph: "To conclude, we found progressive arthropathy..." > replace "adverse event" with "potential adverse event".

Response:

We changed in the text:

“To conclude, we found progressive arthropathy… and radiologists should be aware of this potential adverse event.”
